# Notifiable Disease Surveillance and Practicing Physicians

**DOI:** 10.3201/eid1103.040361

**Published:** 2005-03

**Authors:** Gérard Krause, Gwendolin Ropers, Klaus Stark

**Affiliations:** * Robert Koch Institute, Berlin, Germany

**Keywords:** Surveillance, infectious diseases, physicians, Germany, survey, reportable diseases, policy review

## Abstract

Primary care physicians in Germany are essential participants in infectious disease surveillance through mandatory reporting. Feedback on such surveillance should reflect the needs and attitudes of these physicians. These issues were investigated in a questionnaire survey among 8,550 randomly sampled physicians in Germany in 2001. Of the 1,320 respondents, 59.3% claimed not to have received any feedback on infectious disease surveillance, and 3.7% perceived feedback as not important. Logistic regression analysis showed that physicians in the former East Germany were 2.2 times more likely to have received feedback than those in the former West Germany. Physicians preferred to receive occasional reports (e.g., in case of outbreaks, 31.6%) as opposed to actively having to search for constantly updated information on the Internet (7.8%). The preferred formats were fax (31.7%), mail (30.9%), and the official organ of the German Medical Association (*Deutsches Arzteblatt*) (30.5%). Feedback of surveillance data to physicians should be delivered through occasional nonelectronic reports on current issues of local public health importance.

## Abstract 2

Surveillance data should be delivered to physicians through occasional nonelectronic reports on current issues of local public health importance.

In most countries, notifiable disease surveillance systems rely on mandatory reporting of cases by physicians and laboratories. Although primary care physicians are likely to remain the first and most qualified entry post into such an information system, little research is available on the knowledge, attitudes, and needs of these physicians regarding surveillance of reportable diseases.

In 2001, a new infectious disease control law (IfSG) was implemented in Germany. Most diseases are to be reported to the local county (Landkreis) health department from where they are reported by the state (Land) to the national surveillance institute (Robert Koch Institute). The IfSG also introduced national case definitions for notifiable diseases.

The aim of this study was to identify the needs and attitudes of primary care physicians towards public health surveillance. Studies in various countries have concluded that low compliance of physicians with notification systems is partly caused by insufficient feedback of surveillance data to the physicians (*1*–*3*). However, practical information is lacking on how this feedback should be organized. This study identified ways to accommodate the needs of physicians regarding surveillance to increase their notification compliance.

## Methods

We conducted a survey among primary care physicians in Germany identified by the Green Cross, a non-profit, nongovernmental organization that aims to improve health care. We sent a standardized questionnaire to a random sample (N = 8,550, 14.5%) of 60,280 primary care physicians to be returned by mail on a voluntary and anonymous basis (*4*).

The questionnaire included the following items: sociodemographic and practice-related characteristics, information about changes in the IfSG, expectations from the surveillance system, and reporting practices. West German States were defined as the States of former West Germany including Berlin. The questionnaire was pilot-tested for usefulness and validity by 70 primary care physicians.

Univariate statistical analysis, chi-square tests, and t-tests were used as appropriate. We performed multivariate logistic regression to identify predictors for the level of feedback received. Variables were kept in the model according to the likelihood ratio statistic (forward selection p < 0.05). SPSS software version 11.0 (SPSS Inc., Chicago, IL, USA) was used for statistical analysis.

To assess response bias, the mail survey was followed by a telephone survey. We conducted this survey with 14.5% of the primary care physicians who had originally been sampled for the mail survey. We asked a limited set of questions on the demographic characteristics of the respondent. The data were compared with responses on the completed questionnaires to identify possible bias due to nonresponders.

## Results

Overall, 1,320 completed questionnaires (response rate 15.4%) were returned and included in the study. Of all participating physicians, 87.6% resided in the former West Germany and 86.0% in urban areas. Other demographic characteristics are shown in Table 1. Two-thirds of the physicians saw 250–750 patients per month (median 500 patients per month). Internet access was available for 67.4% of the responders.

**Table 1:** Demographic characteristics of primary care physicians in Germany and the study participants, 2001

Characteristic	All physicians in Germany* (N = 59,610), %	Study population (N = 8,550), %	Survey (questionnaire) (N = 1,320), %	Survey (telephone) (N = 656), %
Age, y ≤39 40–49 50–59 ≥60	13.7 40.3 35.0 11.0	– – – –	10.6 38.7 40.9 9.8	6.1 32.5 43.5 18.0
Sex Male Female	68.3 31.7	70.6 29.4	73.9 26.1	72.8 27.2
Specialization General medicine Internal medicine Other	73.2 26.8	70.7 28.0 1.3	72.6† 24.9 2.5	75.6 22.9 1.5
Region West† East	83.3 16.7	88.6 11.4	83.9 16.1	84.0 16.0

*According to the National Association of Statutory Health Insurance Physicians. †Including Berlin.

Of the physicians, 47.9% felt sufficiently informed about the new infectious disease law. A specific need for information was expressed for the following items: rights and duties of the physician with respect to reporting (55.5%), criteria for disease notification (43.7%), which diseases are reportable (38.4%), rights and duties of the local health department (36.3%), notification format (33.2%), who in the public health system is responsible for outbreak investigations and control measures (33.1%), confidentiality issues (17.1%), and other aspects (4.5%). The existence of case definitions was unknown to 86.5% of the respondents; 75.2% expressed the desire to have case definitions available.

Only 40.7% of the physicians had received any feedback on surveillance data. In the former East Germany, a higher proportion of the physicians received feedback, compared with those in the former West Germany (56.7% vs. 38.8%; p < 0.001). Multivariate logistic regression showed that physicians in the former East Germany were 2.19 times more likely to have received feedback on surveillance data than their colleagues in the former West Germany (95% confidence interval 1.54–3.10).

The sources from which the physicians had received feedback (537 responders) were scientific literature (58.1%), daily press (20.9%), the weekly Epidemiological Bulletin of the Robert Koch Institute (18.4%), reports of the state health departments (11.9%), and other sources (15.3%). Table 2 shows the results of the questions related to how the physicians would like to receive feedback.

**Table 2:** Feedback preferences for epidemiologic notifiable disease surveillance data for primary care physicians in Germany, 2001 (N = 1,320)

Preference	%
Preferred time for feedback (maximum of 1 response)	
Quarterly	33.4
Semiannually	27.2
Upon an occasion (e.g., outbreak)	31.6
Access through the Internet*	7.8
Preferred region on which to deliver feedback (maximum of 2 responses)	
Own county (Kreis)	41.1
Own state (Bundesland)	29.3
Germany	34.8
Europe	28.8
Worldwide	37.3
Preferred medium by which to deliver feedback (maximum of 2 responses)	
Fax letter	31.7
Fax call	2.0
Mail	30.9
Official organ of the German Medical Association (Deutsches Arzteblatt)	30.5
E-mail	20.9
Internet	16.2
Weekly national epidemiologic bulletin	16.5
***Preferred issues for feedback*** (multiple responses)	
Outbreak of infectious diseases	85.2
Trends	47.6
Control of vaccination programs	31.5
Revelation of imported or travel-associated diseases	60.4
Recommendations on preventive measures	65.3
Other	1.4

*Constant availability of information for active retrieval through the Internet by user.

The average monthly time invested in disease reporting was 1.17 hours (median 1, range 0–48) under the new law, compared with 1.02 hours (median 1 hour, range 0–48, p < 0.001) under the old surveillance system. Primary care physicians in the former East Germany invested more hours (median 1, mean 1.92, range 0–48) compared with their colleagues in the former West Germany (median 1, mean 1.08, range 0–30), but the difference was not significant (p = 0.07). Half (50.2%) of the participants stated that under the current system, the obligation to report diseases is likely to influence their diagnostic approach compared with 41.1% under the old surveillance system (p < 0.001).

Of the physicians, 73.9% expressed their willingness to participate in voluntary infectious disease sentinel projects (75.5% in the former West Germany vs. 66.2% in the former East Germany, p < 0.05). The following criteria were chosen when asked about the 2 most important conditions for voluntary participation: easy handling (77.7%), feedback of results (43.1%), and financial compensation (38.9%).

For the nonresponder analysis, a sample of 1,241 physicians (14.5%) was chosen from the 8,550 to whom the questionnaires had been originally sent. Of those 1,049 physicians who could be reached by telephone, 656 (62.6%) agreed to participate. Responders differed significantly from nonresponders for the following items: responders were younger (mean age 49.4 years vs. 51.6 years among the nonresponders; p < 0.001), more often specialists of various medical practices rather than general practitioners (11.8% vs. 5.6%, p < 0.001), and were less likely to know about the special rules that exclude costs for infectious disease laboratory diagnosis from budgetary limitations (74.5% vs. 79.0%, p = 0.03). No differences existed between responders and nonresponders for any other variables.

## Discussion

The survey revealed some unexpected findings about attitudes and expectations of primary care physicians toward the notifiable infectious disease surveillance system. Only half of the respondents felt sufficiently informed about the new law. Studies in southern Africa (*5*), Australia (*1*), and the United Kingdom (*6*) have also found that the list of notifiable diseases is not well known by physicians. This underscores the need to repeatedly inform physicians about the notifiable disease surveillance system.

The survey showed that physicians request information on the occurrence of outbreaks in their county or other acute issues with direct implications and that they are hesitant to actively retrieve the information on the Internet or through faxing (where the user dials the fax server number of a provider to receive a fax). This finding is compatible with findings in the United Kingdom and Australia that general practitioners make limited use of computers (*7*–*9*). A possible consequence may be that local health departments maintain a fax mailing list of all relevant physicians in their county and distribute concise warnings about important public health events as they occur. During a major measles outbreak in northern Germany in 2001, this method was implemented very successfully (*10*).

The finding that physicians in the former East Germany spend more time on notification and receive feedback more often than their colleagues in the former West Germany is consistent with the results of a survey among health departments, in which health departments in the former East Germany would write their own local surveillance reports significantly more often than health departments in the former West Germany (*11*). These observations suggest that the public health system in the East German Democratic Republic had a stronger emphasis on infectious disease surveillance and reporting than in the West German Federal Republic. These differences have been major enough to remain detectable >10 years after unification of West and East Germany. Whether similar differences will be detected in the European Union between western European member states and eastern member states that have recently joined the Union should be determined.

One major goal of the new infectious disease control law was to drastically reduce the number of diseases reportable by the physician to increase notification compliance (*12*). However, this goal may be wishful thinking since the physicians claimed that time invested in notifications had increased slightly in the new system.

Whether the obligation to report certain diseases influences the diagnostic behavior of physicians has rarely been addressed. Half of the respondents in our survey stated that this was indeed the case. This influence may depend on the situation and the disease. Clinical diagnoses of diseases perceived by physicians to be of little importance may be less likely to require microbiologic testing to avoid the administrative task of notification (*13*). Conversely, diseases may be considered important because they are notifiable, thus resulting in a higher probability of microbiologic testing of samples.

The physicians related their willingness to participate in sentinel systems not so much on the financial compensation or the feedback of such activities but rather on the easy handling of the reporting procedure. In this context, it seems to be a priority to generate compatible interfaces between the software systems increasingly used by general practitioners that allow easy generation of disease reports to health departments. Within a national initiative for electronic-government, the Robert Koch Institute is currently developing a concept for highly automated Internet-based reporting of infectious diseases for physicians and laboratories.

Although the response rate of 15% is not unusually low for a survey among physicians, those who participated may have been particularly interested in infectious disease and public health surveillance, thus leading to a bias towards a greater willingness to comply with the system and to be interested in feedback. However, analysis of the nonresponders did not find any evidence for a relevant selection bias. Physicians’ use of media may differ between countries and also change over time, and the situation may be different for participants of research networks or sentinel surveillance systems.

This study showed that the feedback of surveillance data to primary care physicians should use conventional, nonelectronic formats and concentrate on current outbreaks or other public health issues directly relevant to the physician. Low physician compliance with reporting has led to intensification of laboratory-based reporting systems in which electronic notification systems appear to be easier to implement (*1*,*9*,*14*–*17*). However, laboratory-based surveillance systems only include diseases that are confirmed by a complete laboratory diagnosis. Physician notification should take place at a much earlier stage of the diagnostic process, enabling local health departments to rapidly initiate control activities. However, at times of increasing cost awareness in healthcare systems, the proportion of infectious diseases confirmed by laboratory diagnosis may decrease further. Parallel to implementing syndromic systems to achieve early and sensitive surveillance, improving classic disease notification systems for physicians (*18*) will also be important.
